# Immunosensing for Early Detection of Rheumatoid Arthritis Biomarkers: Anti-Cyclic Citrullinated Peptide Antibodies Based on Tilted-Fiber Bragg Grating Biosensor

**DOI:** 10.3390/bioengineering10020261

**Published:** 2023-02-16

**Authors:** Hsin-Yi Wen, Chia-Chin Chiang, Rou-Yu Chen, Wei-Zhi Ni, Yu-Qiao Weng, Yao-Tsung Yeh, Hsiang-Cheng Hsu

**Affiliations:** 1 Department of Chemical and Materials Engineering, National Kaohsiung University of Science and Technology, Kaohsiung 80778, Taiwan; 2 Department of Mechanical Engineering, National Kaohsiung University of Science and Technology, Kaohsiung 80778, Taiwan; 3 Department of Medical Laboratory Science and Biotechnology, Fooyin University, Kaohsiung 83102, Taiwan

**Keywords:** tilted-fiber Bragg gratings (TFBGs), self-assembled sensor layer, cyclic citrullinated peptides (anti-CCP), immunosensing

## Abstract

Rheumatoid arthritis (RA) is regarded as a chronic, immune-mediated disease that leads to the damage of various types of immune cells and signal networks, followed by inappropriate tissue repair and organ damage. RA is primarily manifested in the joints, but also manifests in the lungs and the vascular system. This study developed a method for the in vitro detection of RA through cyclic citrullinated peptide (CCP) antibodies and antigens. The diameter of a tilted-fiber Bragg grating (TFBG) biosensor was etched to 50 μm and then bonded with CCP antigens and antibodies. The small variations in the external refractive index and the optical fiber cladding were measured. The results indicated that the self-assembled layer of the TFBG biosensor was capable of detecting pre- and post-immune CCP antigen and CCP peptide concentrations within four minutes. A minimum CCP concentration of 1 ng/mL was detected with this method. This method is characterized by the sensor’s specificity, ability to detect CCP reactions, user-friendliness, and lack of requirement for professional analytical skills, as the detections are carried out by simply loading and releasing the test samples onto the platform. This study provides a novel approach to medical immunosensing analysis and detection. Although the results for the detection of different concentrations of CCP antigen are not yet clear, it was possible to prove the concept that the biosensor is feasible even if the measurement is not easy and accurate at this stage. Further study and improvement are required.

## 1. Introduction

Rheumatoid arthritis (RA) is a common autoimmune disease characterized by chronic inflammation of the synovial joint [1,2,3,4]. Under most circumstances, RA results in the development of pannus, which ultimately leads to joint damage [5]. Genetically predisposed people who develop RA gradually lose their self-tolerance and begin to produce autoantibodies during this long-term process [5,6]. A phase of asymptomatic autoimmunity is concomitant with a phase of illness risk, characterized by prototypic autoantibodies that are reactive against post-translationally modified proteins, generally cyclic citrullinated peptides (CCPs), which are citrullinated antigens [7,8,9,10]. Carriers of these antibodies to modified protein antigens can be asymptomatic for years and even decades [11,12]. Finally, some people enter a new phase in which symptoms of synovitis appear. It has been demonstrated that early diagnosis alongside proactive use of disease-modifying antirheumatic drugs is conducive to the course of the disease [13,14]. The transition of immune health to autoimmunity and ruthless tissue inflammation over decades is consistent with the progressive remodeling of the immune system. RA is now regarded as a decades-long (if not lifelong) disease, with phases that are distinct in time, space, and pathogenesis. A series of pathogenic procedures caused by a pivotal defect or causative antigen that ultimately leads to joint inflammation have not been identified [15,16]. For this reason, early and accurate diagnosis has become of the utmost importance. Current detection methods are as follows: K. C. Ramos et al. developed a simple straight-channel microdevice and CCP conjugated magnetic nanoparticles (MNPs-CCP) as solid support for quantifying anti-CCP [17]. The dynamic range of concentration was 0.70 to 2000 U/mL^−1^. J. Ma et al. developed a simple label-free electrochemical immunosensor for the detection of anti-CCP-ab constructed using nitrogen-doped graphene (N−G) and gold nanoparticles (AuNPs) [18]. The dynamic range of concentration was 0.125 to 2000 pg/mL^−1^. T.-H. Wu et al. developed magnetic beads surface-coated with fragment-crystallizable regions of immunoglobulin G and biotinylated CCP, enabling the detection of these two RA biomarkers within 55 min with only 5 μL of serum [19]. However, most of the testing methods require a relatively high technical threshold or a longer testing time.

The field of Optical Fiber (OF) has been extensively researched since 1970. The use of optical fibers has expanded from optical transmission waveguides for telecommunications to different sensors and devices, namely for monitoring temperature, mechanical strain, refractive index (RI), pressure and measuring concentration [20,21,22,23]. The above indicates that optical fibers can be used in various fields, from environmental monitoring to biomedical diagnostics and food safety, and that optical sensors are relatively easy to develop and simple to manufacture, such as FBG and TFBG [24,25,26,27,28,29]. Optical fiber-based biosensors feature a simple and compact design that enables online monitoring and seamless collection and analysis of big data [30,31,32,33,34]. These sensors could potentially be used as sensor layers for collecting big data in health monitoring [35,36,37]. In recent years, optical fiber-based biosensors have received widespread attention for their applicability in the rapid detection of various biological parameters. Several types of optical fiber-based biosensors have been developed. In 2012, Sachin K. Srivastava’s team [38] proposed a localized surface plasmon resonance (LSPR) fiber optic glucose sensor, which detects glucose oxidase (GOx) at concentrations from 0 to 250 mg/dL by attaching glucose oxidase to AuNP in the sensing region. In 2016, a research team led by Tuan Guo [39] proposed a fiber optic biomedical sensor with a nano-grade silver coating with a thickness of 20–30 nm on TFBG to stimulate the SPR phenomenon and use it to examine urinary protein changes in nephropathy, comparing healthy, diseased, and treated groups. In 2020, Ali Najafzadeh [40] performed in vitro experimental tests to investigate the behaviors and applications of gratings attached to the intact and fractured thighbone, and the results showed that it was important to configure effective arrangements and orientations of FBG sensors with respect to fracture position and fix the implant for future in vivo experiments. In 2021, Hsin-Yi Wen [41] and colleagues developed a tilted-fiber Bragg grating (TFBG) biosensor for sensitivity analyses and parameter control during the manufacturing process. The surrounding refractive index (SRI) of the cladding mode and the mechanical sensing of the Bragg and ghost modes in the biochemical cross-sensitivity analysis were controlled by processing parameters such as the rotating tilt angles of the phase mask, the duration of laser exposure dose, and the reducing fiber diameters. The results of the study indicated that a 10° tilt angle and a five-minute exposure resulted in a greater cross-sensitivity capacity in mechanical sensing, while also obtaining TFBGs with broad spectral coverage in the cladding and Bragg modes at the same time. In 2022, Sanzhar Korganbayevet al. [42] investigated the effect of etching on the RI sensitivity of TFBGs. The diameter of the optical fiber was etched from 125 µm to 13 µm using hydrofluoric acid. After each etching step, the TFBGs were calibrated using RI solutions with two ranges. The optimal loss sensitivities of the unetched TFBGs for high and low RI ranges were 1008 dB/RIU and 8160 dB/RIU, respectively. At a high RI range, the optimal wavelength sensitivity was 38.8 nm/RIU at an optical fiber diameter of 100 μm; at a low RI range, the optimal wavelength sensitivity was 156 nm/RIU at an optical fiber diameter of 40 μm. Additionally, the study examined the effect of etching on the spectral intensity of the cladding modes, their wavelength separation, and sensor linearity. The study provided the optimal etching parameters for obtaining the best sensitivity, light intensity, and fiber thickness configuration. In 2018, Qi Wang and colleagues [43] proposed a surface plasmon resonance (SPR) TFBG biosensor based on graphene oxide (GO) and staphylococcal protein A (SPA) for detecting human immunoglobulin G (IgG). The gold film on the sensor surface was first immobilized with GO and then modified with SPA to increase the sensor’s sensitivity. Due to its large specific surface area and large number of functional groups, GO was capable of absorbing more antibodies. The combination of SPA and the fragment crystallizable (FC) region of anti-body molecules enables the extension of the fragment antigen-binding (Fab) region, such that highly oriented antibodies are immobilized on the sensor surface, thus increasing the antigen–antibody binding efficiency. The experimental results demonstrated that the GO-SPA-modified TFBG-SPR biosensor had a sensitivity of 0.096 dB/(μg/mL) and a limit of detection (LOD) of 0.5 μg/mL. In comparison with TFBG-SPR biosensors modified using GO or SPA alone, the GO-SPA-modified TFBG-SPR biosensors responded better to human IgG solutions with a concentration range of 30–100 μg/mL. In 2022, Bowie Zhou and colleagues [44] proposed a voltammetry sensor connected with iron oxide nanoparticles (IONPs) through bifunctional aldehyde amine linkers for the immunosensing of RA biomarkers. Voltammetry sensing was employed to develop an immunosensor on an interdigitated electrode (IDE), and a high immobilization of the CCP probes was achieved through green synthesized IONPs. The IONPs were immobilized with a probe on the IDE through bifunctional aldehyde–amine linkers. Due to the increased CCP occupancy and the highly efficient electric transfer of IONPs, higher changes in the current could be observed when the CCPs were bound with the anti-CCPs. The results revealed that in the linear range from 8 to 250 pg/mL, the sensitivity and LOD of the anti-CCPs were 8 and 15 pg/mL, respectively, while the regression coefficient were R^2^ = 0.9637. In this study, a portable, compact, simple, and user-friendly TFBG-based optical fiber sensing platform for rapid CCP detection through multiple self-assembled optical fiber sensing layers and anti-CCP-modified protein reactions was developed. An optical fiber biosensor was fabricated to detect CCP reactions and obtain concentration-specific pre- and post-immunosensing spectral signals corresponding to the CCP reactions. Antigen–antibody reactions were performed through silylation; activation of 3-Mercaptopropionic acid (3-MPA), 1-ethyl-3-(dimethyl aminopropyl) carbodiimide hydrochloride (EDC), and N-hydroxysuccinimide (NHS); and the unreacted acid–carbonyl stretching region. Different surface chemistry mechanisms have major impacts on the performance of biosensors, biomaterials, and other surface chemistry derived from them.

In this study, further study and improvement are required. The wavelength shift or the change in the refractive index can be influenced by the large diameter of the TFBG sensor, the thickness of the nanogold layer, and the unevenness of the self-assembly layer [45,46,47,48,49,50]. Further study will be implemented to address those issues and further improve the performance of the sensor.

## 2. Theory

In Equation (1), below, *n*(*x*,*y*) is a function representing the refractive index perturbation induced by the gratings on the cross-section of an optical fiber [46]:

(1) $$\Delta n\left(x,y\right)=\Delta n\cos\left[\left(\frac{4\pi }{\Lambda }\right)\left(z\cos\left(\theta \right)+y\sin\left(\theta \right)\right)\right]$$

where *x* and *y* are the components of the cladding mode electric field, Λ is the wavelength period, *x* and *y* is the Bragg grating tilt angle. The sole difference between TFBGs and FBGs is that the tilt plane of the gratings alters the input core mode and the coupling coefficients between the guided modes and the radiation modes in the entire structure. As shown in Equation (2), the coupling coefficient is calculated by the transverse electric field component of the mode under consideration [51]:

(2) $$\kappa =C\iint {}_{-\infty }^{\infty }{\vec{E}}_{\mathrm{core}}^{*}\cdot {\vec{E}}_{r}\Delta n\left(x,y\right)dxdy$$

where *C* is a constant of proportionality related to the normalization of the transverse mode fields ($E_{core}$ and $E_{r}$) and Δn(x.y) is the function representing the refractive index perturbation due to the grating in the cross-section of the fiber.

**Figure 1:**
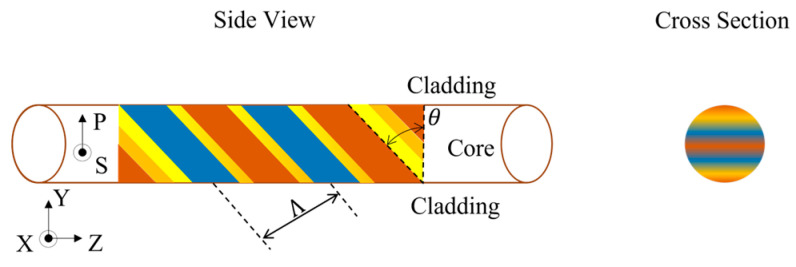
Side and cross-section views of the index perturbation modes caused by the tilted gratings in an optical fiber.

Integration is performed on the x-y cross-section of an optical fiber. In FBGs, grating perturbation is often a constant in the integration domain (the core). In TFBGs, the index perturbation mode [51] is chosen, as shown in Figure 1.

Equation (1) yields the energy field, which is equivalent to the loss variation [51].

## 3. Materials and Methods

### 3.1. Fabrication of TFBG Fiber Sensors

**Figure 2:**
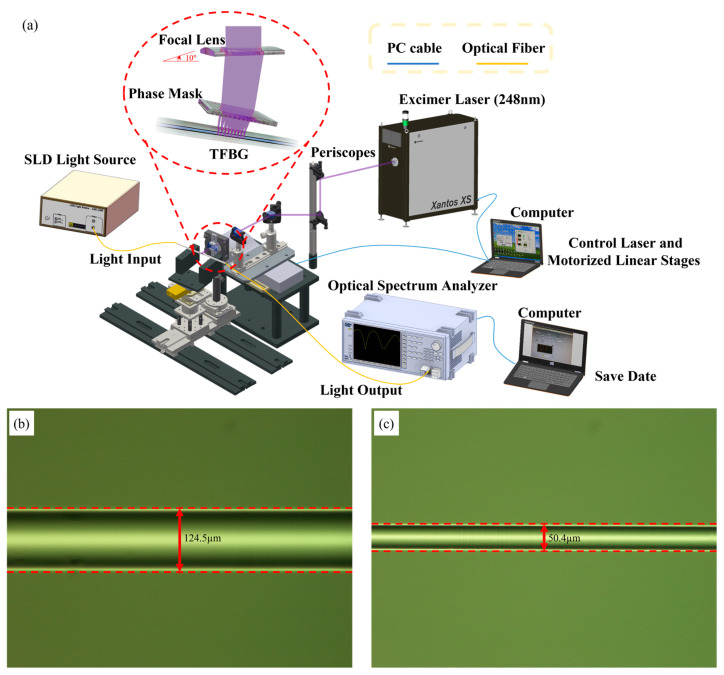
(**a**) Experimental setup for TFBG fabrication; (**b**) Optical microscope image of the unetched TFBG sensor; (**c**) Optical microscope image of the etched TFBG sensor.

TFBGs are FBG-based microstructures with short periods. Optical fibers are transversely irradiated using a krypton fluoride (KrF) excimer laser (Xantos XS 500; COHERENT, Coherent Inc., Saxonburg, PA, USA at a wavelength of 248 nm. Microstructures were etched within the core using a phase mask (O/E Land Inc., Albany, NY, USA). In this study, a special boron-doped photosensitive fiber (PS1250/1500, FIBER CORE, Fibercore, Southampton, UK) was applied for processing. When the KrF excimer laser was reflected by three mirrors and then focused through a planoconvex lens, the phase mask was placed 2 to 5 mm in front of and away from the focal point, and perturbations were formed when the laser exited the phase mask. The variation of the refractive index in the core at the center of the scope of processing marked a periodic distribution that formed gratings. The setup for fabricating TFBGs is shown in Figure 2a. The unetched TFBG is shown in Figure 2b.

Because the resonance of the cladding mode depends on the effective refractive indexes of the core and cladding, reducing the cladding diameter of the optical fiber changes and increases the fiber’s sensitivity to the external refractive index. In this study, after the buffer was removed from the photosensitive optical fiber, the scanning TFBG sensors were fabricated, and wet etching was performed at a constant temperature using a buffered oxide etch (BOE) [52]. The temperature of the etch solution is associated with the etching speed. A higher temperature produces a faster etching rate and vice versa. The experiments were carried out at a constant temperature of 30 °C, while the fiber diameter was reduced at a constant speed of 0.5 μm/min. The diameter of the optical fiber sensor was etched from 125 to 50 μm. The wet-etched optical fiber is shown in Figure 2c.

### 3.2. Self-Assembly of the Nanosensor Layer of TFBGs

During the self-assembly of the biomedical coating layer of the TFBGs, Piranha solution was produced by mixing one part of hydrogen peroxide (H_2_O_2_, 30%, Sigma-Aldrich, St. Louis, MO, USA, Merck Pty. Ltd., Darmstadt, Germany, an affiliate of Merck KGaA, Darmstadt, Germany) with three parts of sulfuric acid (H_2_SO_4_, 98%, Sigma-Aldrich, Merck Pty. Ltd., an affiliate of Merck KGaA, Darmstadt, Germany). The sensor was placed into a quartz glass tube (TUBE ART NO. 34502–99, procured from Kimble) and then immersed in Piranha solution transferred through a syringe needle for 10 min until hydrophilic hydroxyl groups (-OH) formed on the surface. The sensor was then immersed in 10 wt% 3-Mercaptopropyl trimethoxysilane (MPTMS, Sigma-Aldrich, Merck Pty. Ltd., an affiliate of Merck KGaA, Darmstadt, Germany) and methanol for 24 h and then dried at 100 °C. The sensor was then placed into a quartz glass tube and then immersed in nanogold particles (AuNP, 10 nm, Sigma-Aldrich) Merck Pty. Ltd., an affiliate of Merck KGaA, Darmstadt, Germany for two hours. AuNP makes the sensor generate a phenomenal localized surface plasmon resonance (LSPR) phenomenon.

**Figure 3:**
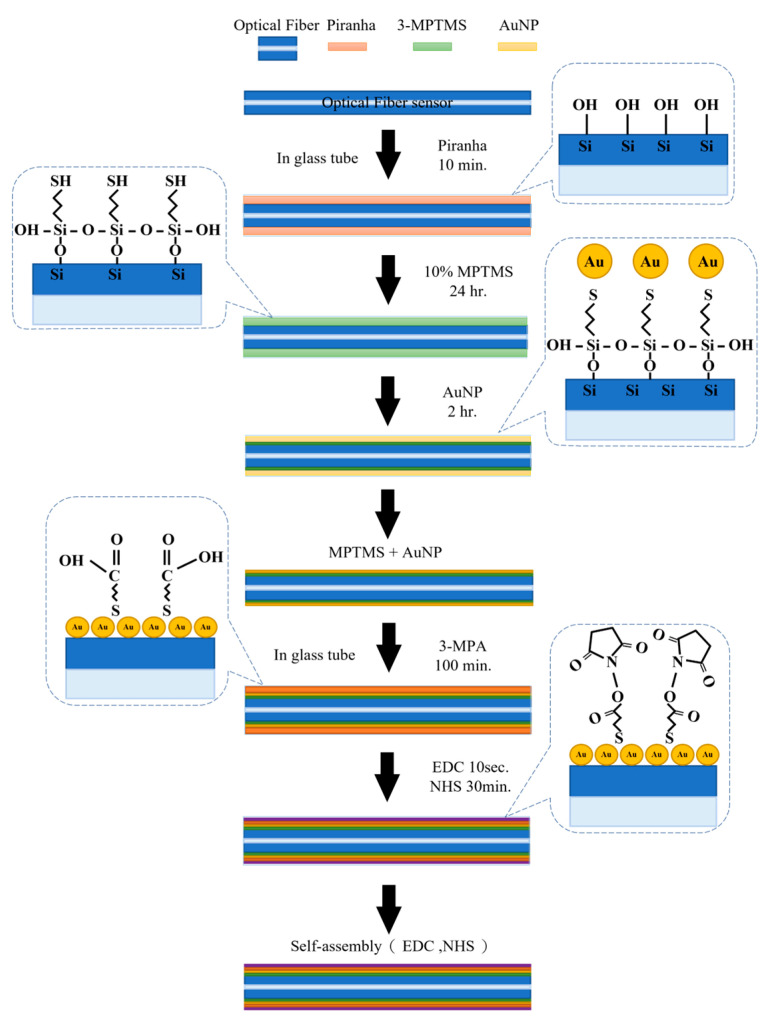
Experimental procedures for piranha solution etching, gold nanoparticle, 3-MPA, and EDC/NH self-assembly.

The sensor was immersed in 10 wt% 3-MPA(Sigma-Aldrich, Merck Pty. Ltd., an affiliate of Merck KGaA, Darmstadt, Germany) and methanol solution for two hours, to complete the self-assembly of 3-MPA. The sensor was then cleaned with methanol (Sigma-Aldrich, Merck Pty. Ltd., an affiliate of Merck KGaA, Darmstadt, Germany) and phosphate-buffered saline (PBS, Uni-Onward Co, New Taipei City, Taiwan) to remove non-bonded 3-MPA molecules. Next, the sensor surface was activated using EDC/NHS (Sigma-Aldrich, Merck Pty. Ltd., an affiliate of Merck KGaA, Darmstadt, Germany) [53,54] for better antigen attachment when 3-MPA was activated. Lastly, the sensor was cleaned with DI water and PBS. The experimental procedure is shown in Figure 3.

### 3.3. Analysis of Fiber Surface

**Figure 4:**
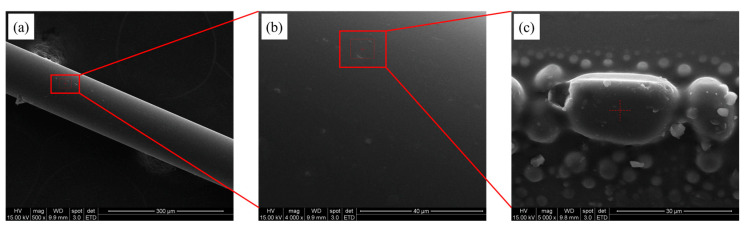
SEM images of the surfaces of the self-assembled layers of the optical fiber at (**a**) 500 times magnification; (**b**) 4000 times magnification; (**c**) 5000 times magnification with 3-MPA magnification.

The morphology of the optical fiber surface of the modified matrices was characterized by scanning electron microscopy (SEM, FEI Quanta 200, Graz, Austria), as shown in Figure 4. Before SEM, the surface of the sensor was coated with carbon. In Figure 4a–c, the self-assembled layer of the fiber was photographed using magnifications of 500×, 4000×, and 5000×, respectively. In addition, the scale bar represents microns in Figure 4a–c. It can be seen that the self-assembled sensing layer is attached uniformly to the surface of the fiber. In Figure 4b,c, AuNP particles in the size range of 10–30 μm can be found on the sensing layer surface.

### 3.4. CCP Detection through the TFBG-Based Biosensor

**Figure 5:**
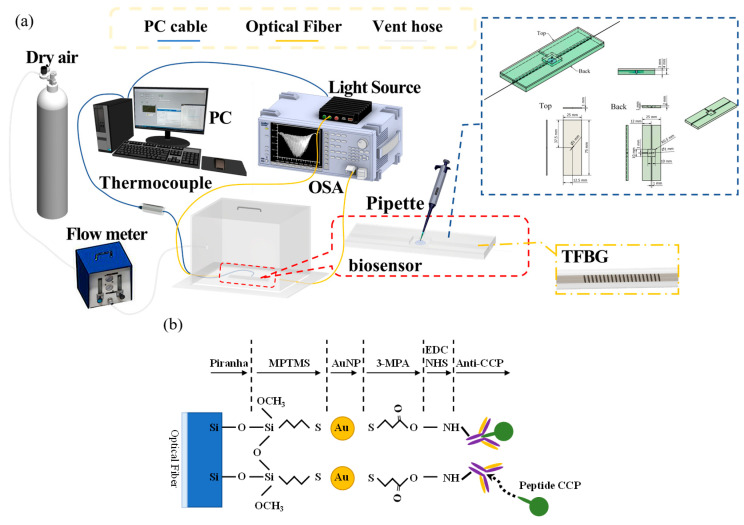
(**a**) Experimental setup of CCP detection through the TFBG sensor; (**b**) reaction mechanism of the formation of chemical bonds on the TFBG sensor surface following EDC/NHS activation.

In the first phase, the objective was to perform detections by changing the anti-CCP levels and immobilizing the antigens. The sensor was immersed in bovine serum albumin, (BSA, Uni-Onward Co, New Taipei City, Taiwan) and PBS at a ratio of 1:1000. Acetic acid stripping buffer was prepared by mixing 10 mL PBS with 1 mL acetic acid (AC) and 0.1 mL emulsifier (Tween80, Shun Ching Raw Material Co., Ltd., Kaohsiung City, Taiwan). The self-assembled TFBG sensor was placed into a microfluidic platform which can inject liquid to be tested and fixed sensor with one end connected to an optical spectrum analyzer (OSA, MS9740A, Anritsu Company, Inc, Atsugi-shi, Kanagawa, Japan) and the other connected to a superluminescent LED source (SLED, DL-BP1 5169A, DenseLight, Singapore), as shown in Figure 5a. Ten minutes after PBS was injected into the microfluidic channel, anti-CCP (PBS: Anti-CCP = 1:1) was injected and the spectral changes were recorded every 15 s for 30 min. BSA was then injected into the sensor region and the spectral changes were recorded for 30 min and the unbonded BSA was removed using PBS. PBS was injected into the microfluidic channel, followed by antigen solution (PBS: CCP = 1:1) 10 min later, and the spectral changes were recorded every 15 s for 30 min. After the process was completed, the sensor was immersed in a stripping buffer (HiMedia Laboratories Pvt. Ltd., Maharashtra, India) for 5 min, and then cleansed twice with PBS for 10 min to separate the antigens and antibodies and to allow the binding of new antigens. PBS was injected into the microfluidic channel, followed by CCP antigen solution (PBS: CCP = 1:1) 10 min later, and the spectral changes were recorded every 15 s for 30 min. The sensor was cleansed with acetic acid buffer for 1 min and then twice with PBS for 10 min, to destroy the bonds between the optical fiber and the antibodies. At a fixed antigen concentration of 111 ng/mL, the anti-CCP antibody concentration was sequentially changed from 162.5 ng/mL and 150 ng/mL pre-immune to 180 ng/mL and 187.5 ng/mL immune. Two detection cycles were completed.

In the second phase, the objective was to identify the concentration of reaction-sensitive immune antigens based on the results of the first phase, while changing the anti-CCP levels and immobilizing the antigens. The TFBG sensor was cleansed with Piranha solution for 10 min, and a new sensor layer was coated onto it. The self-assembly procedure of the nanosensor layer outlined in Section 3.2 was carried out, and the binding peptides on the 3-MPA layer were activated using EDC/NHS (as shown in Figure 5b). The crosslinking conditions represent a standard crosslinker solution concentration: EDC/NHS was dissolved in 95% ethanol and stirred for 30 min to produce a homogeneous EDC/NHS (100 mM/100 mM) solution [55]. The TFBG sensor was placed into a microfluidic platform with one end connected to an OSA and the other to an SLED. The antibody solution was injected into the microfluidic channel, and the spectral changes were recorded every 15 s for 30 min. SA was then injected into the sensor region and the spectral changes were recorded for 30 min and the unbonded BSA was removed using PBS. Antigen solution was injected into the channel, and the spectral changes were recorded every 15 s for 30 min. Then, a repeatable biosensor process was carried out in which the sensor was immersed in a stripping buffer for 5 min, and then cleansed twice with PBS for 10 min. The surface of the biosensor was immobilized with immune anti-CCP (1440 ng/mL), and antigen solution was injected into the sensor region of the microfluidic platform at a concentration of 1, 10, 100, and 1000 ng/mL, and the spectral changes were recorded every 15 s for 30 min.

## 4. Results

**Figure 6:**
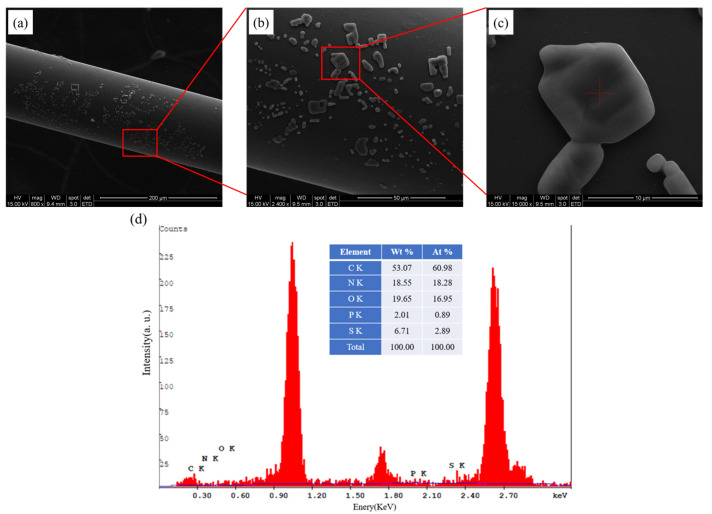
SEM images of the self-assembled layers of the biomedical optical fiber for CCP detection at (**a**) 800 times magnification; (**b**) 2400 times magnification; (**c**) 15,000 times magnification; (**d**) EDS component analysis of the self-assembled layers of the biomedical optical fiber for CCP detection.

The SEM images of the bonded antibody–antigen samples on the optical fiber sensor surface are shown in Figure 6. Before SEM, the surface of the sensor was coated with carbon. In Figure 6a–c the self-assembled layer of the fiber was photographed using magnifications of 800, 2400, and 15,000, respectively, and the scale bar represents microns in Figure 6a–c. The particles on the surfaces under different magnifications in Figure 6b,c, the mean particle size was 5.794 μm, and the particle is antigen–antibody amino acids consisting of O, N, P, S, and other elements. The results of the EDS element analysis of the surface coating of the optical fiber are shown in Figure 6d. It is demonstrated that the amino acid elements N and P of the antibodies and antigens be found on the optical fiber surface.

**Figure 7:**
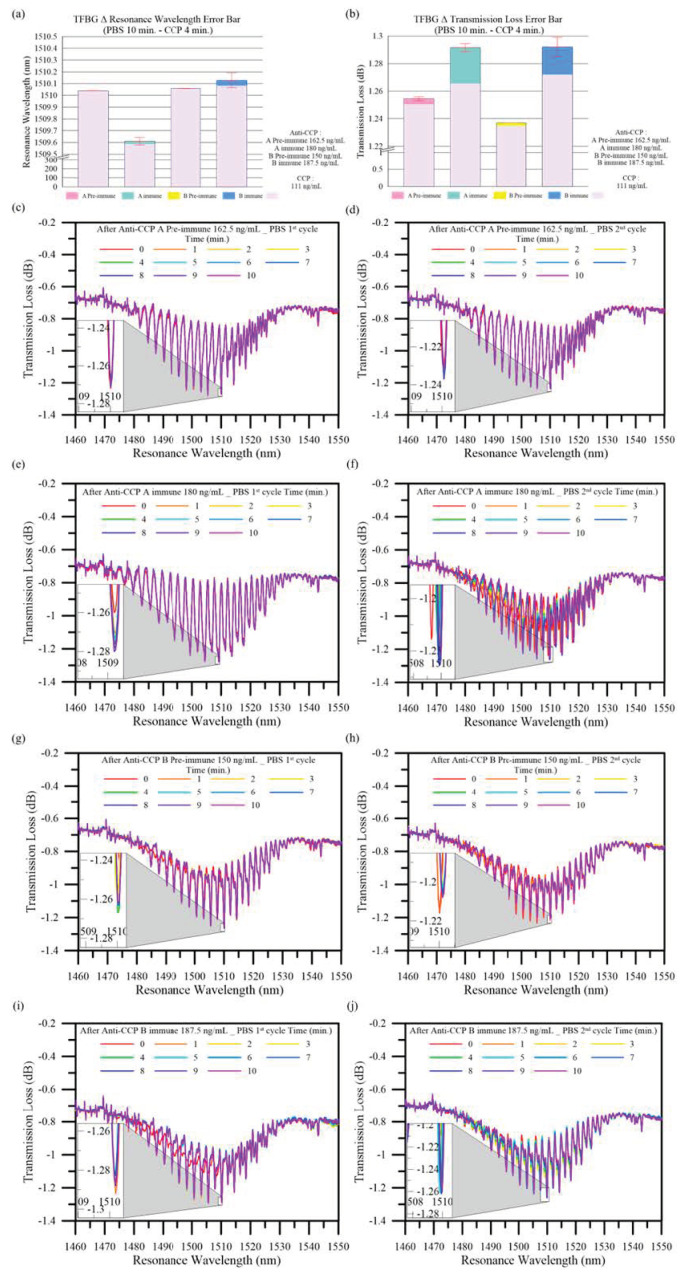
The mean variation and standard deviation in the (**a**) resonant wavelength shift and (**b**) transmission loss detected by the TFBG sensor after 10 min PBS immersion and 4 min CCP immersion. (**c**,**d**) Pre-immune CCP concentration of 162.5 ng/mL 1 cycle and 2 cycle spectrum graphs, respectively. (**e**,**f**) Pre-immune CCP concentration of 180 ng/mL 1 cycle and 2 cycle spectrum graphs, respectively. (**g**,**h**) Pre-immune CCP concentration of 150 ng/mL 1 cycle and 2 cycle spectrum graphs, respectively. (**i**,**j**) Pre-immune CCP concentration of 187.5 ng/mL 1 cycle and 2 cycle spectrum graphs, respectively.

The spectral changes were recorded within 1 to 30 min following antigen injection, and the variations in the resonant wavelength shift and the transmission loss are shown in Figure 7c–j. After two detection cycles had been compared and analyzed, four minutes after antigen injection and the 10th minute after PBS injection, the mean and standard deviation of the variations in the wavelength shift and transmission loss were determined, as shown in Figure 7a,b. A and B are separated from peptides whose terminals are carboxylic acid functional groups and amino groups. Since the antibodies are cultivated from different strains of rabbits, the concentration is slightly different, but still approaching the concentration between 150–170 ng/mL analyzed under control. The mean antigen resonant wavelength shift was 0 nm +/− 0.1 nm at a pre-immune CCP concentration of 162.5 ng/mL. The mean transmission loss was 0.004 dB +/− 0.001 dB. The mean antigen resonant wavelength shift was 0.022 nm +/− 0.023 nm at an immune CCP concentration of 180 ng/mL. The mean transmission loss was 0.026 dB +/− 0.003 dB. The mean antigen resonant wavelength shift was 0 nm +/− 0.0 nm at a pre-immune CCP concentration of 150 ng/m. The mean transmission loss was 0.002 dB +/− 0.002 dB. The mean antigen resonant wavelength shift was 0.045 nm +/− 0.064 nm at an immune CCP concentration of 187.5 ng/mL. The mean transmission loss was 0.020 dB +/− 0.007 dB. At different concentrations, the post-immune solutions generated spectral reactions. At a CCP concentration of 162.5 ng/mL, there was a 6.5-fold difference in the mean transmission loss between pre- and post-immunity. At a CCP concentration of 150 ng/mL, there was a 10-fold difference in the mean transmission loss between pre- and post-immunity. The spectrum variations match Equation (1).

**Figure 8:**
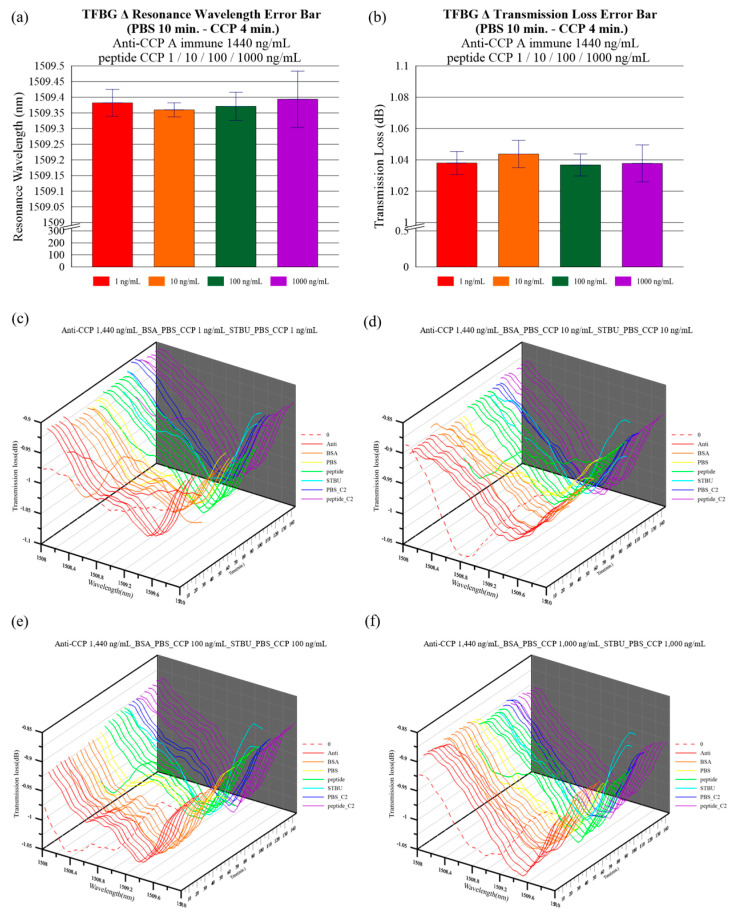
The mean variation and standard deviation in the (**a**) resonant wavelength shift and (**b**) transmission loss detected by the TFBG sensor after 10-min PBS immersion and 4-min CCP antigen immersion. (**c**–**f**) Measurement spectra for CCP antigen concentrations of 1 ng/mL, 10 ng/mL, 100 ng/mL, and 1000 ng/mL, respectively.

The variation in the antigen concentration was analyzed in the second phase of this study, and 3D spectrum graphs are shown in Figure 8c–f. The means and standard deviations of the wavelength shift and transmission loss are shown in Figure 8a,b. The mean spectral variation was obtained 4 min after antigen injection. The mean antigen resonant wavelength shift was 0.034 nm +/− 0.043 nm at a CCP antigen concentration of 1 ng/mL. The mean transmission loss was 0.012 dB +/− 0.007 dB. The mean antigen resonant wavelength shift was 0.011 nm +/− 0.023 nm at a CCP antigen concentration of 10 ng/mL. The mean transmission loss was 0.017 dB +/− 0.009 dB. The mean antigen resonant wavelength shift was 0.022 nm +/− 0.045 nm at a CCP antigen concentration of 100 ng/mL. The mean transmission loss was 0.011 dB +/− 0.007 dB. The mean antigen resonant wavelength shift was 0.045 mm +/− 0.09 nm at a CCP antigen concentration of 1000 ng/mL. The mean transmission loss was 0.012 dB +/− 0.012 dB. While the wavelength shift and the transmission loss are small, the mean antigen resonant wavelength shift has 0.011 nm and the transmission loss has 0.017 dB at a CCP antigen concentration of 10 ng/mL.

**Figure 9:**
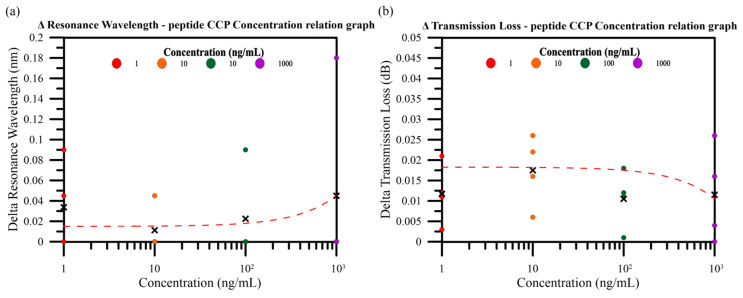
Relationship between CCP antigen concentration and the variation in the (**a**) resonant wavelength shift and (**b**) transmission loss.

As shown in Figure 9, we used the relationship depicted in Figure 8c–f between CCP antigen concentration and the variation to generate the graphics, whereby the mean resonant wavelength shift increased as the CCP antigen concentration increased from 10 ng/mL to 1000 ng/mL. Additionally, the variation in the mean transmission loss increased when the CCP antigen concentration increased from 100 ng/mL to 1000 ng/mL. By comparing Figure 9a,b, it was found that sometimes the spectral wavelength did not change. However, changes do occur to the transmission loss. This is an optical fiber biosensor suing the intensity modulation principle excited by light loss where the dominant effect of localized surface plasmon resonance (LSPR) depends on the thickness of the nanogold layer. Thin-film materials and thickness will cause a significant difference between the resonance wavelength and loss of the low wavelength and the long wavelength part. For nanoparticles, LSPR occurs at the typical plasmonic metal-dielectric interface. The biosensor was manufactured based on a TFBG, and works by the intensity-modulation principle excited by changes in the refractive index that is attenuated by LSPR. Therefore, the inhomogeneity of the optical sensor surface and the thickness of the nanogold layer will have negative impact on the stability of the wavelength shift, leading to relatively large error in transmission loss. We also compiled the relevant literature on the use of fiber optic sensors to detect CCP, the results of which are presented in Table 1 [44,56,57,58]. From Table 1, it can be found that the sensor has a good detection range from 1 to 1000 ng/mL.

In this study, attention was focused on the monitoring of light field intensity of the sensor. The main objective was to prove that the concept of the biosensor is feasible even if the measurements are not easy or accurate at this stage. Further study and improvement are required. The wavelength shift or the change in the refractive index can be influenced by the large diameter of the TFBG sensor, the thickness of the nanogold layer and the unevenness of the self-assembly layer. Further study will be performed to address those issues and further improve the performance of the sensor.

## 5. Conclusions

In this study, a self-assembled TFBG biomedical sensor was shown to be capable of detecting CCP antigen concentrations within four minutes. In the first phase of the study, comparative pre- and post-immune detections were carried out at a fixed antigen concentration of 111 ng/mL. In Sample A, the antigen concentration was changed from 162.5 ng/mL pre-immune to 180 ng/mL immune; in Sample B, the antigen concentration was changed from 150 ng/mL pre-immune to 187.5 ng/mL immune. The results showed that both Samples A and B had immunospecific reactions. The variation in the mean transmission loss of post-immune Sample A was 6.5 times greater than that of pre-immune Sample A. The variation in the mean transmission loss of post-immune Sample B was 10 times greater than that of pre-immune Sample B. This demonstrates that the self-assembled TFBG biomedical sensor was reactive toward anti-CCP identification. In the second phase of the study, given that the post-immune reactions were experimentally validated in the first phase, the CCP antibody concentration was fixed at 1440 ng/mL, while the antigen concentration varied from 1, 10, 100, and 1000 ng/mL. This study developed a TFBG sensor with a self-assembled detection layer that allows the specific detection of CCPs. Detections are quick and simple and are achieved by loading and releasing the test samples. This study marks a novel advancement in medical immunosensing analysis, and had a good detection range from 1 to 1000 ng/mL. This study focused on the monitoring of the light field intensity of the sensor. The wavelength shift or the change in the refractive index can be influenced by the large diameter of the TFBG sensor, the thickness of the nanogold layer, and the unevenness of the self-assembly layer. Further study will be performed to address those issues and further improve the performance of the sensor. The results indicate that, although the results for the detection of different concentrations of CCP antigen are not yet clear, it is possible to prove the concept that the biosensor is feasible even if the measurements are not easy or accurate at this stage. Further study and improvement are required.

## Figures and Tables

**Table 1:** CCP biomedical immunoassay literature compilation.

Authors	Immunoassay/Sensor	LOD	Linear Range	Year	Ref.
B.T. Nguyen et al.	CE-LIF	0.1 μg/mL	N/A	2018	[56]
S. Guerrero et al.	SPCdEs	2.5 IU/mL	10–1000 U/mL	2020	[57]
C.-Y. Lin et al.	ELISA	0.16 IU/mL	0.25–1500 IU/mL	2022	[58]
B. Zhou et al.	Voltammetry Sensor	15 pg/mL	8–250 pg/mL	2022	[44]

## Data Availability

The data is unavailable due to privacy.
